# USING A LARGE LANGUAGE MODEL TO CLASSIFY DEMENTIA CAREGIVERS’ HEALTH INFORMATION WANTS

**DOI:** 10.1093/geroni/igae098.0353

**Published:** 2024-12-31

**Authors:** Zhuochun Li, Zhimeng Luo, Ning Zou, Daqing He, Bo Xie, Robin Hilsabeck, Alyssa Aguirre

**Affiliations:** University of Pittsburgh, Pittsburgh, Pennsylvania, United States; University of Pittsburgh, Pittsburgh, Pennsylvania, United States; University of Pittsburgh, Pittsburgh, Pennsylvania, United States; University of Pittsburgh, Pittsburgh, Pennsylvania, United States; The University of Texas Austin, Austin, Texas, United States; The University of Texas Health Sciences Center San Antonio, San Antonio, Texas, United States; The University of Texas Austin, Austin, Texas, United States

## Abstract

Many caregivers of persons living with dementia use social media to express dementia care-related health information wants (HIWs), i.e., types of information caregivers seek that can help them manage challenges and concerns. Recent developments in large language models (LLMs) hold promise in automatic classification of HIWs expressed in large quantities of social media posts. As a first step, we evaluated GPT-3.5 Turbo’s performance in classifying 60 social media posts based on our previously developed HIW framework, which includes 7 first-level categories, including daily care. Under daily care, there are 7 second-level categories and a total of 32 third-level subcategories. All 60 posts were previously verified by 3 experienced clinicians as being in the daily care category; 40 posts were in the second-level category of mood and behavior management and 20 in the second-level category of safety; and 20 posts in each of the 3 third-level categories: agitation; memory loss and confusion; and driving safety. We asked GPT-3.5 Turbo to classify the posts on the first level; 14 (23%) were accurately classified to daily care. Next, we asked GPT-3.5 Turbo to classify those 14 posts into one of the second-level categories in daily care; 10 (71%) were accurately classified into mood and behavior management or safety. Finally, we asked GPT-3.5 Turbo to classify the 10 remaining posts into the third-level subcategories. Seven (70%) were accurately classified: agitation 5/5 (100%), driving safety 2/2 (100%) and memory loss and confusion 0/3 (0%). Disappointed by GPT-3.5 Turbo’s performance, we discuss plausible reasons and solutions.

